# Book Notices

**Published:** 1868-02

**Authors:** 


### Book Notices.

Obstetric Clinic: A Practical Contribution to the Study of Obstetrics and the Diseases of Women and Children. By GEORGE T. ELLIOT, Jr., A.M., M.D., Professor of Obstetrics and Diseases of Women and Children in the Bellevue Hospital Medical College; Physician to the Bellevue Hospital, and the New York Lying-In Asylum; etc., etc., etc. New York: D. APPLETON & Co. 1868.

This is an octavo volume of 458 pages, published in excellent style; paper and type both being good. It embraces seventeen chapters, on the following topics: Relations of Albuminuria to Pregnancy; Prophylaxis of Puerperal Eclampsia—varieties of puerperal convulsions; Chloroform and Venesection in Puerperal Eclampsia; Relation of Epilepsy to the Puerperal State—Puerperal Mania; Ante-Partum Hemorrhage; Induction of Labor; Effect of the Tonic Circular Contraction of a Band of Uterine Muscular Fibres on Labor—Brow and Face Presentations—Rupture of the Uterus; Post-Partem Hemorrhage; Obstetric Operations in Deformed Pelves; Choice, Use, and Applications of Forceps; Embryotomy; Version; Inflammatory Complications in the Surgical Treatment of the Diseases of Women; Certain Conditions of the Bladder in Women; Dangers from Compression of the Funis; Recto-Perineal Abscess; Kiesteine.

The author has had ample opportunities for clinical observation, and his work will be found of real value to the general practitioner. For Sale by S. C. GRIGGS & Co. Price, $4.50.

---

A Practical Treatise on the Diseases of Children. By D. FRANCIS CONDIE, M.D., Fellow of the College of Physicians; Member of the Amer. Med. Association; etc., etc. Sixth edition, revised and enlarged. Philadelphia: HENRY C. LEA. 1868.

This a full-sized octavo volume of 783 pages, well bound. The former editions have been well known to the reading part of the profession, and generally recognized as one of the best works on the diseases of children in our language. In preparing the present edition for the press, the entire work has undergone a thorough revision by the author. We cordially recommend the work to all, whether students or practitioners. For sale by W. B. KEEN & Co., 148 Lake Street.

---

Pennsylvania Hospital Reports. Vol. I. 1868. Philadelphia: LINDSAY & BLAKISTON.

This is a full-sized octavo volume of 420 pages, published in elegant style. It embraces twenty-three articles, all on topics of direct practical importance, and by the most experienced observers and investigators in the profession of Philadelphia. It is the first of a series of volumes which are promised, by which the important results of hospital experience will become accessible to the profession generally. We have intimations that similar volumes will be issued, founded upon the records of the extensive hospitals of New York and some other cities. The present volume may be found at W. B. KEEN & Co.'s, 148 Lake Street. Price, $5.00.

---

We have only space to acknowledge the receipt of the following works:

Spermatorrhœa: Its Causes, Symptomatology, Pathology, Diagnosis, Prognosis, and Treatment. By ROBERTS BARTHOLOW, A.M., M.D., Prof. Materia Medica and Therapeutics in the Med. College of Ohio; Lecturer on Clinical Medicine, and Physician to the Hospital of the Good Samaritan; etc., etc., etc. Second edition, revised and augmented. New York: WM. WOOD & Co., 61 Walker Street. 1867. Received through W. B. KEEN & Co., 148 Lake St., Chicago.

---

Plastics: A New Classification and a Brief Exposition of Plastic Surgery. A reprint from a report in the Transactions of the Illinois State Medical Society for 1867. By DAVID PRINCE, M.D. Philadelphia: LINDSAY & BLAKISTON. 1868. Pp. 96. Price, $1.50.

---

Diseases of the Heart: Their Diagnosis and Treatment. By DAVID WOOSTER, M.D., Member of the Royal Academy of Medicine and Surgery, Turin; Assistant-Surgeon in the Mexican War; etc., etc., etc. San Francisco: W. H. BANCROFT & Co. 1867. Duo., pp. 210. For sale at No. 113 William Street, New York.

---

Treatment of Diseases of the Throat and Lungs, by Inhalation, with a New Inhaling Apparatus. By EMIL SIEGLE, M.D. Translated from the Second German Edition, by S. NICKLES, M.D. Cincinnati: R. W. CARROLL & Co., 117 West Fourth Street. 1868. Duo., pp. 136.

---

Chronic Diseases of the Larynx, with special reference to Laryngoscopic Diagnosis and Local Therapeutics. By Dr. ADELBERT TOBOLD, Lecturer in the University of Berlin. Translated from the German and edited by GEORGE M. BEARD, A.M., M.D., Lecturer on Nervous Diseases in the Ueiversity of New York. With an Introduction on the History and Art of Laryngoscopy and Rhinoscopy, Rhinitis, Inhalations, and Electrizations applied to Diseases of the Air-Passages; and an Appendix by the Editor. Illustrated by forty-four engravings on wood. New York: WM. WOOD & Co., 61 Walker Street. 1868. Octavo, pp. 279. From W. B. KEEN & Co., 148 Lake Street.

---

Signs and Diseases of Pregnancy. By THOMAS HAWKS TANNER, M.D., F.L.S., Member of Royal College of Physicians, etc. From the second and enlarged London Edition. With four colored plates and illustrations on wood. Philadelphia: HENRY C. LEA. 1868. Octavo, pp. 490. From W. B. KEEN & Co., 148 Lake Street.

